# #MyDepressionLooksLike: Examining Public Discourse About Depression on Twitter

**DOI:** 10.2196/mental.8141

**Published:** 2017-10-18

**Authors:** E Megan Lachmar, Andrea K Wittenborn, Katherine W Bogen, Heather L McCauley

**Affiliations:** ^1^ Department of Human Development and Family Studies Michigan State University East Lansing, MI United States; ^2^ Department of Psychiatry Rhode Island Hospital Providence, RI United States

**Keywords:** social media, depression, community networks, social stigma

## Abstract

**Background:**

Social media provides a context for billions of users to connect, express sentiments, and provide in-the-moment status updates. Because Twitter users tend to tweet emotional updates from daily life, the platform provides unique insights into experiences of mental health problems. Depression is not only one of the most prevalent health conditions but also carries a social stigma. Yet, opening up about one’s depression and seeking social support may provide relief from symptoms.

**Objective:**

The aim of this study was to examine the public discourse of the trending hashtag #MyDepressionLooksLike to look more closely at how users talk about their depressive symptoms on Twitter.

**Methods:**

We captured 3225 original content tweets for the hashtag #MyDepressionLooksLike that circulated in May of 2016. Eliminating public service announcements, spam, and tweets with links to pictures or videos resulted in a total of 1978 tweets. Using qualitative content analysis, we coded the tweets to detect themes.

**Results:**

The content analysis revealed seven themes: dysfunctional thoughts, lifestyle challenges, social struggles, hiding behind a mask, apathy and sadness, suicidal thoughts and behaviors, and seeking relief.

**Conclusions:**

The themes revealed important information about the content of the public messages that people share about depression on Twitter. More research is needed to understand the effects of the hashtag on increasing social support for users and reducing social stigma related to depression.

## Introduction

Seven out of 10 Americans use social media to share personal information, engage with content, and connect with others [1]. One of the most popular platforms, Twitter, has over 313 million active users who produce 500 million tweets—140-character comments—each day [2,3]. Hashtags (ie, a word or phrase preceded by a # sign) are often used in tweets to signal a specific topic and link tweets together by topic, thereby facilitating the potential for dialogue on a topic. Twitter has become a valuable resource to study topics ranging from diurnal sleep patterns to dietary behavior and changes in mood [4-6]. Twitter users commonly tweet in-the-moment experiences of daily life, with one of the most common topics consisting of emotional status updates, which may not be feasible to share in real-world, face-to-face settings [7]. Therefore, Twitter offers a unique data source for examining stigmatized topics such as mental health. Whereas researchers have only recently begun studying Twitter data, prior examinations of user- and investigator-initiated hashtags such as #Depression, #Schizophrenia, #DearMentalHealthProfessionals, and #WhyWeTweetMH [8-10] have already illustrated the value of doing so.

Depression is one of the most prevalent mental health issues and is a disorder well known to carry social stigma [11,12]. Individuals with depression are more likely to become socially isolated and less likely to seek help [12,13]. Social rejection–related stressors are significantly linked to the cognitive, emotional, and biological changes that lead to depression [14]. Fearing social interactions may lead to loneliness, which is also a risk factor for depression [15]. In one study, individuals who reported more social and emotional support were 87% less likely to report recurrent depression [16]. Connection through engaging in a Web-based community such as Twitter may foster social support among those experiencing depression, as well as provide a potential resource for relief. Sharing or engaging with others online may buffer the fear of in-person social interaction and provide a platform for normalizing the discussion about this prevalent mental health issue.

Prior research indicates that social media can provide a supportive environment for talking about depression [17]. An analysis of responses to an investigator-initiated Twitter hashtag intended to ask users why they tweet about their mental health found that users primarily post about their mental health to seek connection with other users [8]. In one study, Facebook users who received online reinforcement from friends about a post that indicated depressive symptoms were subsequently more likely to discuss symptoms outwardly on Facebook [18]. Further, having at least one characteristic in common, such as depression, can foster a sense of community between members in a Web-based group [19]. Although social media can provide a supportive environment, it may also have deleterious effects. Social media can become a modality fraught with negative social comparison, jealousy, and comment wars, which can decrease quality of life [20-24]. Studies have found associations between social media use and depression [25,26], social isolation and loneliness [27], poorer sleep quality and lower self-esteem [28]. In one study examining the relationship between social media use and mood and personality disorders, those who spent more time on Facebook were more likely to have clinical symptoms of depression [29].

Many of the previous studies of depression-related social media content have relied heavily on Facebook [18,23,29,30], whereas fewer have studied Twitter content [9,10]. However, findings indicate that there are key differences between the two platforms that lead to uniquely different content. Twitter users frequenly use pseudonyms and are more likely to be connected with users they have never met in person, offering users a more anonymous way to communicate. Studying discourse on Twitter may provide a less biased account of individuals’ experiences, as it is naturalistic, contains a broad population of people who may not frequently participate in research, and is often anonymous, thus overcoming some limitations of traditional data collection methods.

Given the potential for understanding stigmatizing disorders through Twitter [30], it is imperative to look more closely at the discourse on Twitter about mental health. To start with, studies are needed that qualitatively examine how those with depression talk about their symptoms online. As May is mental health month, several hashtags on mental health such as #MyDepressionLooksLike, #MyAnxietyLooksLike, and #MyMentalIllnessFeelsLike trended in May of 2016. The hashtag #MyDepressionLooksLike caught the attention of popular news sites, such as the *Huffington Post*, *ABC news*, *US News* and *World Report*, *NY Magazine*, *Teen Vogue*, and *Cosmopolitan*, and attracted users. In the first known study of this hashtag, we analyzed tweets from #MyDepressionLooksLike using a content analysis approach, to answer the following research question: how do people talk about their experiences of depression on Twitter using the hashtag #MyDepressionLooksLike?

## Methods

To improve our understanding of discourse on social media about depression and public communication of symptomology, we downloaded tweets using the hashtag *#* MyDepressionLooksLike from the social networking site Twitter. We used NCapture, an addition to the qualitative analysis NVivo software (QSR International), to collect tweets. NCapture allowed for reliable access to Twitter’s public streaming application programming interface (API) [31] and retrieved a 10% random sample from Twitter’s public content. Data collection spanned 1 week, from Wednesday, May 25, 2016 to Wednesday, June 1, 2016. Data were not collected over Memorial Day weekend, as the holiday would skew regular computer use. Data were captured at approximately 10:00 AM Eastern Standard Time each weekday. Out of a total of 11,178 tweets that were captured, 9237 were retweets. For this sample, we restricted analysis to the 3225 original content tweets. Each observation included the following: username, number of followers, number following, tweet ID (a number assigned to the tweet by Twitter’s API), text of tweet, date and time tweeted, tweet URL, and latitude-longitude data of each Internet Protocol address. Only tweets that directly referenced #MyDepressionLooksLike were included in this analysis. Although tweets often included pictures, only text was included in the final dataset. Tweets without relevance were removed from this dataset [32]. Content was limited to English-language tweets. After eliminating public service announcements, spam, and tweets with links to pictures or videos, our final dataset included 1978 tweets.

As Twitter provides a public platform for users to interface, is widely accessible, and is among the most used social media sites, *tweets should be considered public conversation* [32]. Furthermore, the anonymity allowed by Twitter, in combination with the creation of virtual communities and simulated social interaction, makes Twitter an optimal setting to examine the effects of anonymity on public discourse surrounding mental health, patient needs, and symptomology. Researchers Bruckman [33] and Whitehead [34] establish that this analysis meets the standards to waive informed consent and similar guidelines, based on the public nature of Twitter content. It is important to note that tweets can only be captured if the Twitter user’s profile is set to public, thus protecting those who have private profiles from being subject to research studies. This study was approved by the (university name blinded for review) institutional review board.

### Analysis

Data were imported into NVivo and analyzed by the first 2 authors using qualitative content analysis methods. Both coders have expertise in clinical depression. Content analysis is a systematic method for making inferences from text to summarize the content of communication [35]. In this study, an inductive open coding approach was used to allow themes to be generated directly from the data [36]. The 2 independent researchers read the first 10% of the tweets to become familiar with the content. Then, each researcher independently categorized each of the first 10% of the tweets into codes and subcodes, as is common in sensitizing concepts of qualitative research [37]. The researchers met to compare codes and to establish consensus on the definition of each code. All 1978 tweets were then read independently by each researcher and sorted into the defined codes, a process designed to establish trustworthiness and credibility and to consider whether new codes emerged in the remaining tweets [35]. The coders then discussed the coding of each tweet until consensus was reached. No new themes emerged when the full dataset was coded. In some cases, tweets fit into more than one theme. In these cases, we either chose the theme most fitting or coded the tweet for more than one theme. The codebook is available from researchers upon request.

### Twitter Users

Although we were unable to gather demographic data from the Twitter users, we were aware of what Twitter users look like in general. Twitter users are vastly more overrepresented in populous counties, according to a study that compared Twitter users to the US Census data [38]. The proportion of men and women who use Twitter is roughly equivalent [39]. Twitter users tend to be younger than 50 years and have a college education [40]. Trends also indicate a higher rate in the number of users who earn high incomes and identify as white [39,40].

**Figure 1:**
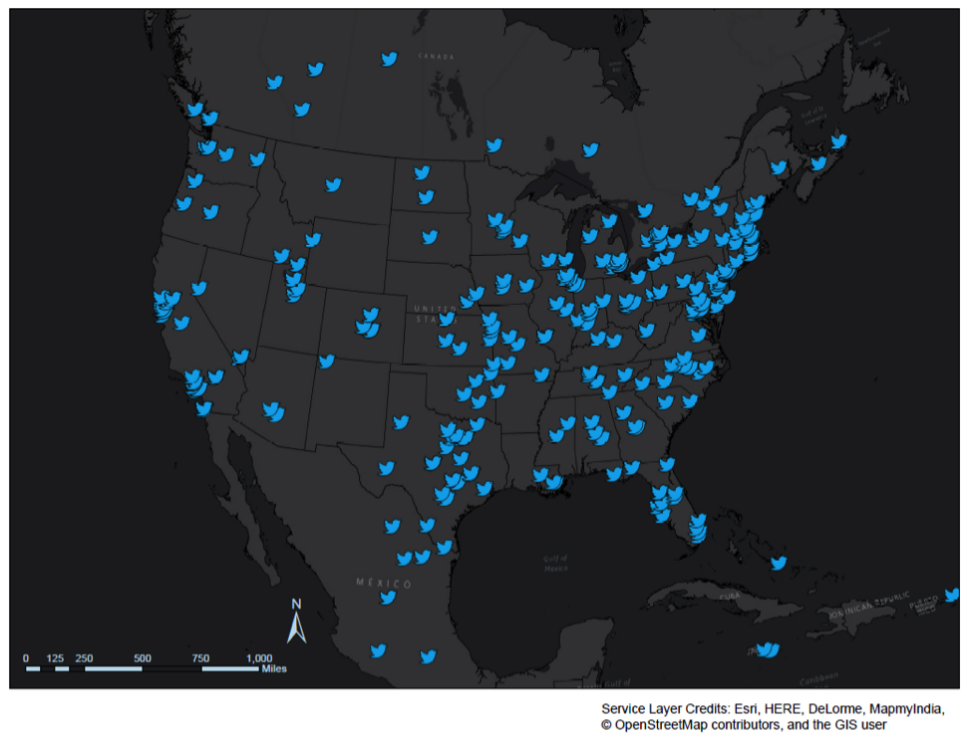
Map of Twitter icons representing tweet locations of users.

Although the majority of Twitter users live in urban areas, Web-based communities may be particularly useful in rural communities for those experiencing health symptoms that carry stigma [41]. In this context, rural social media users are more frequently the recipients of support regarding health topics, whereas urban users are providers of social support [41]. In this study, we were able to obtain geotags of users, which included the latitude-longitude location of the user at the time they tweeted. We used this information to assess the geographic location of the sample. We used the geotag instead of the profile location because about 34% of Twitter users enter fake locations in their profile [42]. An image of the geographic location of tweets can be seen in Figure 1. The majority of tweets in the final dataset came from North America; therefore, only those are shown in the figure.

## Results

Seven themes emerged from the #MyDepressionLooksLike Twitter data: dysfunctional thoughts, lifestyle challenges, social struggles, hiding behind a mask, apathy and sadness, suicidal thoughts and behaviors, and seeking relief. See Table 1 for an overview.

### Theme 1: Dysfunctional Thoughts

Tweets in this theme described distorted and dysfunctional cognitions (n=498 tweets) and included negative thoughts about self; perceptions that feelings of depression are invalid; feeling unlovable, hopeless, helpless, and invisible; as well as putting others’ needs before their own. Some Twitter users described difficulty with their own thoughts:

I can’t bear to lie awake with my thoughts.

...being unable to enjoy alone time, because the inside of my head is too dark of a place.

Tweets also revealed negative views about oneself such as:

...standing in the mirror and repeating “I hate myself” every other week.

...a punch to the gut that makes me curl up in bed because I’m a worthless and disgusting trash excuse for a human.

Many tweets described these negative views of oneself in the form of anger at themselves, as described by one user:

...incredible anger, self-loathing and unfounded guilt, every morning I wake up.

Twitter users also questioned their own feelings of depression:

...feeling like I shouldn’t be depressed bc I have lots of good things in life that I should be happy about.

These negative views about oneself also transferred to views of feeling unlovable by others, as described in the following tweets:

...thinking that everyone hates me, even my friends/family/partner.

...a huge black cloud, drowning, sinking, hopeless. Demons pulling me under. Whispering “just give up, no one loves you.”

...thinking everyone hates me/has a grudge against me when I haven’t done anything to them.

Along with negative views of oneself, hopelessness and helplessness also satiated this theme:

...feeling stuck all the time.

...no options, no choices and no escape.

...internally drowning while everyone else seems to be staying afloat.

Feelings of being invisible also came through in many of the tweets in this category, such as when one Twitter user stated:

...invisible. It’s there but nobody can see.

Finally, putting others’ needs above their own arose throughout the content of this theme, as is described in the following tweet:

...constantly helping people out of their problems and never being able to help myself.

### Theme 2: Lifestyle Challenges

In addition to difficult thoughts and perceptions, lifestyle challenges were often mentioned (n=416 tweets). In this theme, Twitter users described problems with motivation, eating, sleeping, and other daily tasks. A large portion of the tweets in this category revealed difficulty with motivation:

...wanting change but having no motivation to make a change.

...having no motivation to do absolutely anything.

**Table 1:** MyDepressionLooksLike themes and descriptions.

Theme	Description	Sample tweet	Number of tweets
Dysfunctional thoughts	Thoughts about self that are negative, hopelessness, feeling invisible, and unlovable	*feeling eternally unloveable and hopeless*	498
Lifestyle challenges	Difficulty with eating, sleeping, motivation, and daily tasks	*staying in bed at all times because I’ve lost all motivation to do anything*	416
Social struggles	Struggling in social relationships, isolation, loneliness, and feeling like a burden to others	*isolating myself, then getting upset over being lonely. Constantly feeling like I’m annoying or a burden to everyone*	433
Hiding behind a mask	Pretending to be okay in front of others to hide the reality of one’s depression	*a big smile that I wear everyday to hide the sad thoughts inside my head*	236
Apathy and sadness	Expressions of sadness and emptiness	*crying for no reason*	149
Suicidal thoughts and behaviors	Descriptions of self-harm and thoughts of death	*No interest in being awake. There is nothing in me that enjoys being alive. If given an option I’d choose death.*	110
Seeking relief	Both positive and negative means to alleviate the depression; descriptions of self-care as well as coping mechanisms such as alcohol and drugs	*Smiling big & drinking & smoking my problems away to feel numb to the pain* *something I manage with therapy, medication, good habits, and exercise. You can too.*	136

A significant number of Twitter users described difficulty getting out of bed:

...being so mentally exhausted that it turns into physical exhaustion and I literally can’t get out of bed.

...not being able to make it out of the house the entire weekend because leaving bed is an impossible feat.

I think I just spent 36 straight hours in bed.

Parallel to being unable to get out of bed, respondents to the hashtag described a feeling of exhaustion:

Tiredness that is far deeper than muscle fatigue. It’s like my very soul is exhausted.

...being so heavily exhausted that I can’t even take care of myself or anyone/anything else.

Regarding sleeping and eating, users posted the following tweets:

...having no appetite for days on end, constantly hearing “you need to eat” and no one understanding that I can’t.

...being constantly dehydrated because I can’t even get out of bed to get a glass of water.

This also included aspects of hygiene and a lack of self-care:

...not remembering the last time I showered and not caring...being called gross when I tell someone.

Other daily tasks were described as being affected, as described in the following tweets:

...mold growing on dozens of dishes by my bed, not doing laundry for months.

Trouble with school and work was another aspect of this theme:

...a constant fear of failing school #mydepressionlookslike not having any motivation to get my work done.

...my boss telling me I’m not the same person he hired because my happiness is affected (sic) my work.

### Theme 3: Social Struggles

Twitter users reported difficulty with social relationships (n=433 tweets). One Twitter user described it as pushing others away, longing for connection, loneliness, isolation, and feeling like a burden to others. Respondents to the hashtag revealed overall difficulty with social relationships:

...pressing the self-destruct button on relationships because I can’t figure out a way to sustain them.

When describing their social struggles, users often expressed conflicted feelings and behaviors such as wanting closeness but instead pushing loved ones away. One user described this as:

...wanting to just have someone hold me and say it’s ok just one time. But instead yelling at anyone who touches me.

Another Twitter user vividly painted this picture:

...screaming inside—“don’t leave me alone” while simultaneously saying “I’ll be fine.”

This often revealed an underlying fear of judgment from others:

...pushing anyone and everyone away because being alone is the only guarantee of a judgment free space.

In addition to fear of judgment, fear of reaching out and asking for help also came up frequently throughout this theme:

...being scared to reach out to others because how can you make others happy when you can’t even make yourself happy?

These difficulties with social relationships also lead to feelings of isolation and loneliness, as described in the following tweets:

...alienation and feeling so alone in the world that I have no one to talk to.

...feeling disconnected from everyone and everything you ever liked doing while aching for love and belonging.

Often this was something Twitter users seemed to describe as purposefully doing, as described below:

...literally dropping all my friends for months, showing up to school every day in sweats/a hoodie not talking to nobody.

...distancing myself from everyone I love.

Users also described feeling like a burden:

...wanting to socialize and then telling the person I don’t want to see them cause (sic) I feel like a burden.

Respondents to the hashtag also described anger at others in their social relationships:

...anger an outburst because I keep everything inside I can’t trust anyone with my feelings.

### Theme 4: Hiding Behind a Mask

Descriptions of depression were also revealed as being hidden by putting on a mask (n=236 tweets). In this manner, Twitter users described hiding behind a mask to hide the reality of their depression:

always faking a smile and acting like everything is fine when it’s not.

Some respondents to the hashtag described being so good at hiding their depression that they were able to keep it a secret for a long time:

...wouldn’t even know I’ve been battling it for 5 years cause I’m so good at hiding it.

...cause I’d rather put a mask on than let people in to see how vulnerable or broken I am.

This illustrates why Twitter users may feel they need to put on a mask. In addition, Twitter users revealed other reasons, such as:

...a smile to avoid the “what’s wrong” questions.

The tweets in this category also described smiling, laughing, and joking in an effort to mask their depression, as described in the following tweets:

...covered up by a big smile and funny jokes only if they knew about the emptiness within.

...me always joking and laughing to cover up the hurt and suffering that is imbedded within my spirit.

### Theme 5: Apathy and Sadness

Twitter users described symptoms of crying spells and overall sadness and apathy (n=149 tweets). This theme described these emotional effects of what depression looks like. Tweets revealed crying spells and tearfulness. Many tweets described crying when alone:

tears begin to flow when I’m by myself.

Crying seemed to be described consistently throughout this theme:

...crying, crying, crying...oh did I forget to mention crying over EVERYTHING b/c every little thing cuts so much deeper.

Another tweet provides an example of feeling down:

...a “case of the Mondays”; always lurking, waiting to sneak up on me the minute I let my guard down.

In addition to sadness, feelings of emptiness and apathy were also included in this #MyDepressionLooksLike theme. Users tweeted about what depression looks like, describing it as follows:

...a hole carved into my chest and have nothing to fill that inner emptiness.

...feeling completely numb and emotionless.

A sense of the loss of emotion is also included in this emptiness, as indicated by the following tweet:

...the loss of all my former passion and hope.

### Theme 6: Self-Harm and Suicidal Behaviors

Tweets also described self-harm and suicide (n=110 tweets). One individual described self-harm as:

...taking a blade to my skin just to have some sort of evidence of the pain I hold locked up inside.

This way of revealing emotional pain through self-harm was evidenced by another similar tweet:

...lines carved into my skin, to make emotional pain physical, visible.

One Twitter user described the self-harm of depression as:

...long sleeves in the summer time, people staring at my arms and wondering if it’s contagious.

Thoughts of death also persisted throughout this category of tweets, as evident in the following:

...wanting to die 24/7.

...re-editing the same old suicide note.

Wishing for death was another way suicide crept up into these tweets:

...my bed in the darkness with nothing but my thoughts slowly killing me, hoping they will.

Wishing I was in heaven now. Not wanting to live anymore.

### Theme 7: Seeking Relief

Many users described ways to find relief from their depression (n=136 tweets). This included both positive and negative means to alleviate depression, such as descriptions of self-care as well as adverse coping mechanisms such as alcohol and drugs. Finding a way to relieve symptoms, such as receiving professional services, was also an aspect of the tweets in this theme. Negative means to alleviate depression were expressed in the following tweets:

...trying to be productive but watching 5 hours of Netflix (sic) in bed—just so my mind turns off.

...a lot of shopping bags in my closet. I would rather shop the pain away than talk about it.

Other forms of adverse coping skills included alcohol and drugs, as expressed in the following tweet:

...excessive drinking and sleeping because reality sucks.

Medications were also described frequently throughout this theme, as expressed in the tweets below:

...the little green pill that somehow helps me get through every day. For you it’s just life, for me it’s a mission.

...7 medications a day.

In terms of more positive, hopeful ways of seeking relief, one Twitter user described:

And the power of prayer really helped myself to direct my energy towards a calmer path.

Another person tweeted that #MyDepressionLooksLike:

...a child I need to take care of daily—it’s a part of me so I show myself love and kindness, even on the hardest days.

## Discussion

### Principal Findings

Tweets in response to the hashtag #MyDepressionLooksLike saturated various themes describing the inner world of individuals with depression. These themes reveal important information about how people talk about their experiences of depression on Twitter. Some of this included cognitive, lifestyle, and social implications of depression, as well as hiding the reality of it, experiencing sadness, seeking relief, and suicidal thoughts and behaviors.

The themes of this study reflect previous findings on depression. The theme, *dysfunctional thoughts*, included tweets that displayed users’ difficulty with thoughts, such as feeling hopeless and unlovable. Prior literature reveals similar findings, such that people with depression have greater rumination in association with negative material [43,44]. The theme *lifestyle problems* revealed Twitter users’ difficulty with daily activities such as eating, sleeping, exercising, working, and overall motivation. Problems with motivation have also been seen in the literature on depression, such that lack of motivation is considered the number one barrier to employment in adults with a major thought or affective disorder [45]. The *Diagnostic and Statistical Manual of Mental Disorders*, Fifth Edition (DSM-5) lists dysfunctional thoughts and lifestyle problems as symptoms of major depressive disorder (MDD) [13]. Interestingly, engaging on a social media platform such as Twitter may require less energy to connect with others compared with engaging face-to-face. Social media has also been found as a motivator of and barrier to exercise and an influencer of food choices and eating habits [46]. This makes the idea of depression and social media a two-sided coin, creating an easier environment to receive support but also potentially creating barriers to a healthy lifestyle.

In another example, tweets in the social struggles theme often displayed content steeped in isolation, pushing others away, but simultaneously wanting to be close to people. Users also described feeling like a burden, which has been indicated in prior literature [47]. Individuals who feel they are a burden to their family and friends may be more willing to disclose symptoms on an online platform in which they are more anonymous as opposed to Facebook where followers are often your real-world friends [48]. Research indicates that Twitter users are able to talk about mental health openly because they do not fear being judged and are able to vent and feel heard [8]. However, because of the format of Twitter, such as the character limit for tweets, it is possible that it may result in less ability to provide or receive social support compared with other social media outlets. Additionally, sharing negative tweets may lead to co-rumination among users [49]. These online social relationships may replicate in-person relationships with others, providing a much needed connection for those with depression [17]. People with depression or poorer mental health tend to use social media for more social support and emotional connection as opposed to individuals without depression who use it for information sharing [48,50]. It is also unclear whether social connections on Twitter result in the same sense of burden as compared with real-life social ties and how this could influence online communication [47].

Users also tweeted about self-harm and suicidal thoughts and behaviors, paralleling DSM-5 symptoms of MDD [13]. In a previous systematic review of young people using social media for discussing self-harm, the findings suggest that users were supported in the Web-based setting [51]. However, suicide and social media have gained increased attention as news reports reveal harrowing incidents of individuals live streaming their own suicide on sites such as Facebook [52]. Facebook has since initiated a suicide prevention page that offers live-chat support from mental health providers and is testing a suicide program that would use artificial intelligence methods to identify potential suicide or self-injury posts [53,54]. It may be difficult for social media sites to keep up with hashtags because of their ability to generate so much attention in such a short period, making it impossible for self-harm or suicide prevention to adequately take place [55]. However, a Twitter-specific suicide prevention app called the Samaritans Radar designed to detect users who may need help was closed shortly after launching because of vulnerability of those with mental health, including opening this information up to cyber bullies [56]. These prevention attempts reveal potential avenues and controversies of providing support for self-harm and suicide in a Web-based community format. This is particularly important because of the evidence of suicide contagion, in which watching or hearing about suicide increases the likelihood of others engaging in suicidal behaviors [57,58]. Detecting concerns of suicide on Twitter in research raises important ethical questions. Twitter users do not consent to participate and are difficult or impossible to locate, making traditional means of disclosing risk unviable. This is an unresolved ethical dilemma that will need to be addressed as research of Twitter data continues.

Along with potentially providing support, social media platforms can provide guidance for self-management of health problems such as depression [17]. Twitter users may be posting on this hashtag to seek relief from symptoms. In addition to data provided in the description of the seeking relief theme, Twitter users shared information about exploratory treatments undergoing testing, including messages about the magic mushroom compound [59]. Interestingly, research reveals that online community users expect to receive more support than they are willing to provide [60,61]. This may mean their use of social media is a form of seeking relief in the first place. Literature also suggests that the emotional and instrumental support these communities provide actually influences patients’ health states, such as improving from poor to fair outcomes [17]. It may also be that users benefit from sharing mental health experiences online in general, as well as communicating with other users about their experiences. However, in this study, we were unable to analyze these constructs.

### Limitations

Information such as sex, age, relationship status, occupation, socioeconomic status, race, or religion of users was not available, which prevented researchers from drawing study conclusions based on sample characteristics. Furthermore, we were unable to gather information about the potential dialogue occurring between Twitter users, such as retweets and comments, because these retweets often did not include the original hashtag, making them difficult to capture. It is important to note that our sample did not include all tweets associated with the hashtag, making it possible that our data could miss other themes. It is also possible that the brief nature of tweets—140 characters—may limit what we understand about users’ experiences. Finally, posts for the specific hashtag #MyDepressionLooksLike may represent a biased population of Twitter users, such as only those who identify with depression.

### Future Directions

Our study examined the public discourse of depression and depression symptoms on Twitter. Given the role that social support and isolation play in depression and that Twitter removes some barriers for individuals with depression to connect with others, future research is needed to understand the public conversations Twitter users have with one another when users share about their struggles and methods for coping. Given the mixed findings on the effects of social media use, it is also possible that there are deleterious effects of using Twitter to connect on sensitive topics such as depression. It would be useful to understand the benefits and consequences of engaging with the Twitter platform regarding mental health problems. Finally, more research is needed to understand how users’ posts about their depression affect their real-world relationships, given the benefits or consequences this could have for one’s course of illness.

## Abbreviations

API: application programming interface
DSM-5: Diagnostic and Statistical Manual of Mental Disorders, Fifth Edition
MDD: major depressive disorder

## References

[1] (2017). Pew Research Center.

[2] (2017). Internet Live Stats.

[3] (2017). Twitter.

[4] Grinberg N, Naaman M, Shaw B, Lotan G (2013). Semanticscholar.

[5] Hingle M, Yoon D, Fowler J, Kobourov S, Schneider ML, Falk D, Burd R (2013). Collection and visualization of dietary behavior and reasons for eating using Twitter. J Med Internet Res.

[6] Lampos V, Lansdall-Welfare T, Araya R, Cristianini N (2013). Arxiv.

[7] Murthy D, Bowman S, Gross AJ, McGarry M (2015). Do we tweet differently from our mobile devices? a study of language differences on mobile and web-based twitter platforms. J Commun.

[8] Berry N, Lobban F, Belousov M, Emsley R, Nenadic G, Bucci S (2017). #WhyWeTweetMH: Understanding why people use twitter to discuss mental health problems. J Med Internet Res.

[9] Reavley NJ, Pilkington PD (2014). Use of twitter to monitor attitudes toward depression and schizophrenia: an exploratory study. PeerJ.

[10] Shepherd A, Sanders C, Doyle M, Shaw J (2015). Using social media for support and feedback by mental health service users: thematic analysis of a twitter conversation. BMC Psychiatry.

[11] Mathers C (2008). The Global Burden of Disease: 2004 Update.

[12] Barney LJ, Griffiths KM, Jorm AF, Christensen H (2006). Stigma about depression and its impact on help-seeking intentions. Aust N Z J Psychiatry.

[13] American Psychiatric Association (2013). Diagnostic and Statistical Manual of Mental Disorders (DSM-5®).

[14] Slavich GM, O'Donovan A, Epel ES, Kemeny ME (2010). Black sheep get the blues: a psychobiological model of social rejection and depression. Neurosci Biobehav Rev.

[15] Cacioppo JT, Hughes ME, Waite LJ, Hawkley LC, Thisted RA (2006). Loneliness as a specific risk factor for depressive symptoms: cross-sectional and longitudinal analyses. Psychol Aging.

[16] Brinker J, Cheruvu VK (2017). Social and emotional support as a protective factor against current depression among individuals with adverse childhood experiences. Prev Med Rep.

[17] Yan L, Tan Y (2014). Feeling blue? go online: an empirical study of social support among patients. ‎Inf Syst Res.

[18] Moreno MA, Jelenchick LA, Egan KG, Cox E, Young H, Gannon KE, Becker T (2011). Feeling bad on facebook: depression disclosures by college students on a social networking site. Depress Anxiety.

[19] Schwämmlein E, Wodzicki K (2012). What to tell about me? self-presentation in online communities. J Comput-Mediat Comm.

[20] Fox J, Moreland JJ (2015). The dark side of social networking sites: an exploration of the relational and psychological stressors associated with Facebook use and affordances. Comput Human Behav.

[21] Chou HG, Edge N (2012). “They are happier and having better lives than I am”: the impact of using facebook on perceptions of others' lives. Cyberpsychol Behav Soc Netw.

[22] de Vries DA, Kühne R (2015). Facebook and self-perception: individual susceptibility to negative social comparison on Facebook. Personal Individ Differ.

[23] Frison E, Eggermont S (2015). Exploring the relationships between different types of facebook use, perceived online social support, and adolescents depressed mood. Soc Sci Comput Rev.

[24] Radovic A, Gmelin T, Stein BD, Miller E (2017). Depressed adolescents' positive and negative use of social media. J Adolesc.

[25] Schou AC, Billieux J, Griffiths MD, Kuss DJ, Demetrovics Z, Mazzoni E, Pallesen S (2016). The relationship between addictive use of social media and video games and symptoms of psychiatric disorders: A large-scale cross-sectional study. Psychol Addict Behav.

[26] Lin LY, Sidani JE, Shensa A, Radovic A, Miller E, Colditz JB, Hoffman BL, Giles LM, Primack BA (2016). Association between social media use and depression among U.S. young adults. Depress Anxiety.

[27] Yang C (2016). Instagram use, loneliness, and social comparison orientation: interact and browse on social media, but don't compare. Cyberpsychol Behav Soc Netw.

[28] Woods HC, Scott H (2016). #Sleepyteens: social media use in adolescence is associated with poor sleep quality, anxiety, depression and low self-esteem. J Adolesc.

[29] Rosen L, Whaling K, Rab S, Carrier L, Cheever N (2013). Is facebook creating “iDisorders”? the link between clinical symptoms of psychiatric disorders and technology use, attitudes and anxiety. Comput Human Behav.

[30] Jelenchick LA, Eickhoff JC, Moreno MA (2013). “Facebook depression?” social networking site use and depression in older adolescents. J Adolesc Health.

[31] (2017). Twitter.

[32] Cravens JD, Whiting JB, Aamar RO (2015). Why I stayed/left: an analysis of voices of intimate partner violence on social media. Contemp Fam Ther.

[33] Bruckman A (2002). Gatech.

[34] Whitehead LC (2007). Methodological and ethical issues in Internet-mediated research in the field of health: an integrated review of the literature. Soc Sci Med.

[35] Krippendorff K (2012). Content analysis: an introduction to its methodology.

[36] Kondracki NL, Wellman NS, Amundson DR (2002). Content analysis: review of methods and their applications in nutrition education. J Nutr Educ Behav.

[37] Bowen GA (2016). Grounded theory and sensitizing concepts. IIQM.

[38] Mislove A, Lehmann S, Ahn Y, Onnela J, Rosenquist J (2011). Mislove.

[39] (2016). Pew Research Center.

[40] Duggan M, Ellison N, Lampe C, Lenhart A, Madden M (2015). Social media update 2014. Pew Research Center.

[41] Goh J, Gao G, Agarwal R (2016). The creation of social value: can an online health community reduce rural-urban health disparities? Mis Quarterly. MIS Quarterly.

[42] Liu Y, Kliman-Silver C, Mislove A (2014). Mislove.

[43] Joormann J, Gotlib IH (2008). Updating the contents of working memory in depression: interference from irrelevant negative material. J Abnorm Psychol.

[44] Rimes KA, Watkins E (2005). The effects of self-focused rumination on global negative self-judgements in depression. Behav Res Ther.

[45] Braitman A, Counts P, Davenport R, Zurlinden B, Rogers M, Clauss J, Kulkarni A, Kymla J, Montgomery L (1995). Comparison of barriers to employment for unemployed and employed clients in a case management program: An exploratory study. Psychiatr Rehabil J.

[46] Vaterlaus JM, Patten EV, Roche C, Young JA (2015). #Gettinghealthy: the perceived influence of social media on young adult health behaviors. Comput Human Behav.

[47] Hames JL, Hagan CR, Joiner TE (2013). Interpersonal processes in depression. Annu Rev Clin Psychol.

[48] Park M, McDonald D, Cha M (2013). Perception differences between the depressed and non-depressed users in twitter.

[49] Davila J, Hershenberg R, Feinstein BA, Gorman K, Bhatia V, Starr LR (2012). Frequency and quality of social networking among young adults: associations with depressive symptoms, rumination, and corumination. Psychol Pop Media Cult.

[50] Sampasa-Kanyinga H, Lewis RF (2015). Frequent use of social networking sites is associated with poor psychological functioning among children and adolescents. Cyberpsychol Behav Soc Netw.

[51] Dyson MP, Hartling L, Shulhan J, Chisholm A, Milne A, Sundar P, Scott SD, Newton AS (2016). A systematic review of social media use to discuss and view deliberate self-harm acts. PLoS One.

[52] Dwoskin E, Timberg C (2017). The Washington Post.

[53] (2017). Facebook.

[54] (2017). Facebook.

[55] Moreno MA, Ton A, Selkie E, Evans Y (2016). Secret society 123: understanding the language of self-harm on Instagram. J Adolesc Health.

[56] (2017). BBC News.

[57] Cheng Q, Li H, Silenzio V, Caine ED (2014). Suicide contagion: a systematic review of definitions and research utility. PLoS One.

[58] Mueller AS, Abrutyn S (2015). Suicidal disclosures among friends: using social network data to understand suicide contagion. J Health Soc Behav.

[59] Carhart-Harris RL, Bolstridge M, Rucker J, Day CM, Erritzoe D, Kaelen M, Bloomfield M, Rickard JA, Forbes B, Feilding A, Taylor D, Pilling S, Curran VH, Nutt DJ (2016). Psilocybin with psychological support for treatment-resistant depression: an open-label feasibility study. Lancet Psychiatry.

[60] Fox S, Jones S (2007). Pew Research Center.

[61] Preece J, Nonnecke B, Andrews D (2004). The top five reasons for lurking: improving community experiences for everyone. Comput Human Behav.

